# Biochemical and toxicological effects of methanolic extract of *Asparagus africanus* Lam in Sprague-Dawley rats

**DOI:** 10.7717/peerj.9138

**Published:** 2020-06-22

**Authors:** Abubakar El-Ishaq, Mohammed A. Alshawsh, Kein Seong Mun, Zamri Chik

**Affiliations:** 1 Science Laboratory Technology Department, School of Science and Technology, Federal Polytechnic, Damaturu, Yobe State, Nigeria; 2 Department of Pharmacology, Faculty of Medicine, University of Malaya, Kuala Lumpur, Malaysia; 3 Department of Pathology, Faculty of Medicine, University of Malaya, Kuala Lumpur, Malaysia; 4 University of Malaya Bioequivalence Testing Centre (UBAT), Faculty of Medicine, University of Malaya, Kuala Lumpur, Malaysia

**Keywords:** *Asparagus africanus*, Antifertility, Biochemical, Contraceptive, Haematology and toxicity

## Abstract

*Asparagus africanus* Lam. is a plant used traditionally to treat different ailments. Currently, scanty information is available on its safety. The aim of this study is to determine the acute toxicity of the methanolic extract on vital organs and its associated biochemical parameters. Fifteen female Sprague-Dawley rats were divided into five groups. Group I served as normal control, groups II, III, IV, and V were orally administered single dose of crude extract dissolved in distilled water at 5 mg/kg BW, 50 mg/kg BW, 300 mg/kg BW and 2,000 mg/kg BW. Rats were observed for 14 days and body weights were recorded. On day 15, the rats were sacrificed and blood samples were collected for biochemical and haematological analyses, while the liver and kidneys were sampled for histopathological examination. Body weight and haematology parameters results showed significance difference (*p* < 0.05) among means of HGB, RDW, RBC, and MCHC; likewise, (*p* < 0.001) for WBC and platelet among treated groups. Histopathology result showed that kidneys appeared normal while livers were congested with mildly swollen hepatocytes and occasional binucleation. Focal lobular hepatitis was observed in all treated animals. However, hepatic enzymes were not significantly affected and no histopathological harmful effects were observed in kidney*.* In conclusion, methanolic extracts of *A. africanus* are safe up to 2,000 mg/kg BW. The obtained results could be used as a justification for the traditional application of the plant for treatment of various ailments.

## Introduction

There has been a rapid increase in the use of herbal medicines as complements to orthodox medicines. The reasons are ease of access without long waiting times for medical prescriptions, cheaper and lack of regulation by international health authorities on the use of herbal medication (Saad & Said, 2011). Major problems associated with herbal medicines are lack of repository database, adequate standard for its formulation, consistency, safety, quality, and sometimes incorrect identification of herbal materials and pharmacologically bioactive compounds (Ahmad et al., 2006).

There are many plants-derived bioactive constituents of medicinal importance for human and animal health, for instance; traditionally, a mixture of powdered roots of *Cassia occidentalis*, and *Derris brevipes* is used to control female fertility. Preliminary phytochemical studies indicated the presence of steroids, terpenoids, flavonoids and glycosides in the ethanolic extracts of these two plants. These compounds are known to exhibit anti-fertility activity (Badami et al., 2003). Principal chemical compound reported from *Dendrophthoe falcata* for example contains cardiac glycosides, flavonoids and pentacyclic triterpenes. *D. falcata* has been utilized as an anti-fertility agent for women. The aerial part is also used to induced abortion (Pattanayak & Mazumder, 2009).

*Asparagus africanus* Lam belongs to family liliaceae and is widespread in the drier parts of tropical Africa (Hassan et al., 2012) where it has been widely used for medicinal purposes (Bonjar & Farrokhi, 2004). There are many ethno-pharmacological claims on various parts of *A. africanus* (Asfaw et al., 1999), such as treatment of immune disorders (Ahmad et al., 2006), anti-fertility and its health related problems (El-Ishaq, Alshawsh & Chik, 2019a; El-Ishaq et al., 2019b; Geremew, Yalemtsehay & Eyasu, 2006). In addition, its root aqueous extract has been taken regularly as an anti-depressant and to ease childbirth (Hamill et al., 2003), treat hypertension, cancer and epilepsy (Moshi & Mbwambo, 2005), applied externally for treating chronic gout (Lohdip & Tyonande, 2005). The main phytoconstituents that may be responsible for the pharmacological effects of *A. africanus* are saponins, flavonoids and tannins such as spirostanosides, furostanol, sapogenin and lignin (Hassan, Ahmadu & Hassan, 2008). In addition, our previous study confirmed the presence of steroidal saponins such as stigmasterol and sarsasapogenin (El-Ishaq, Alshawsh & Chik, 2019a).

Meanwhile, other studies have reported that the aerial parts of *A. africanus* are used to treat headache, backache, stomach pain, easing childbirth and to stimulate women’s hair growth (Lohdip & Tyonande, 2005; Ribeiro et al., 2010). Batchelor & Scott (2006) reported that shrub and root decoction of *A. africanus* are used in the treatment of stomach disorders. Leaf preparation of this plant had been reported to be potent for management of tuberculosis and as anti-candida. The stem of *A. africanus* is also used for the treatment of tuberculosis (Steenkamp, 2003; Tabuti, Kukunda & Waako, 2010). Its cladodes are used for treating various skin diseases (Giday et al., 2003).

Previous acute toxicity study of the saponin extracts of rhizomes of *A. africanus* in mice has been reported with LD_50_ of 1264.9 mg/kg (Hassan et al., 2012), the anti-inflammatory study of the extract has been shown to be statistically significantly (*p* < 0.05) in decreasing rat paw-oedema when compared to the control (Hassan et al., 2012). On the other hand enzyme-treated *A. africanus* extract has been developed as a novel anti-stress food and dietary supplement (Ito et al., 2014).

Despite all these aforementioned uses of this plant, especially its anti-fertility effects, there is still insufficient data regarding its toxicological effects on vital organs of the body. Therefore, toxicity screening of the root extract of *A. africanus* is of utmost importance (Lipnick et al., 1995). The aim of this study is to determine the acute toxicity of methanolic extract of *A. africanus* (MEAA) on selected organs, via blood chemistry and histopathological analysis using female Sprague-Dawley rats.

## Material and Methods

### Chemicals and drugs

Ketamine hydrochloride and Xylazine were obtained from Parke-Davis (Barcelona, Spain). Methanol was obtained from BDH (London, UK). Eosin and Haematoxylin (Abbey colours). All relevant chemicals were of analytical grade.

### Plant material collection

The root of *A. africanus* Lam plant was collected at kilometre 20 opposite Barema Farm along Nguru road, in Damaturu local government area of Yobe State, Nigeria in September, 2015. This location is at longitude 11°, 44″, 52N, N, latitude 11°, 57″, 35S, E. The plant sample was authenticated by a taxonomist from the Institute of Biological sciences, and sampled plant was assigned voucher number KLU 48696. The plant was then deposited in the Herbarium, University of Malaya. The collected plant was cleaned dust free and air-dried under laboratory condition, before being separately grounded into a fine powder and kept in an air-tight container for further use.

### Procedure of Methanolic extraction

50 g of the fine powder was extracted using 500 mL of methanol, then the procedure was repeated between each extraction to remove the solvent using Rotavapor R-200/205 (Dunn, Turnbull & Sharon, 2004). Each extract was filtered using Whatman filter paper No. 4. The extract was evaporated in vacuum using Buchi Rotary Evaporator (Rotavapor R-200/205) coupled with EYELA Cool Ace (CA-1110) and Buchi vacuum system B-169. The residue was kept in a fridge at 4 °C prior to further analytical use.

### Animal model procurement

This study was conducted using an experimental animal model. Healthy female Sprague-Dawley rats weighing 150–200 g were obtained from the Animal Experimental Unit, Faculty of Medicine, University of Malaya. The rats were randomly distributed into five groups of three (3) rats each and were fed on a diet of standard pellet and tap water. The animals were kept in cages at 25 ± 2 °C temperature and 50 ± 10% humidity, in a standard light/dark cycle (12 h light/12 h dark cycle). All experimental procedures were carried out according to the approval of the Animal Ethics Committee for Experimentation, Faculty of Medicine, University of Malaya, Malaysia (Ethics Reference No. 2015-180505/PHAR/AEI). Furthermore, administration of extract, blood and organs sampling as well as all surgical procedures were carried out with humane care according to Good Practice Guide for the administration of substances and collection of blood, including the routes and volumes (Karl-Heinz et al., 2001).

### Toxicity study

The toxicity test was conducted using a slightly modified method of Kadagi et al. (2014); and (OECD, 2001). Fifteen female Sprague-Dawley rats were randomly distributed into five groups of three rats each. The animals were selected and assigned with permanent marker to permit individual identification, kept in their cages for at least seven days with close observation prior to dosing to allow for acclimatization to the laboratory conditions and for detection of any noticeable abnormal behaviour. The rats were fasted for 4 h before administration of extract, but water was allowed *ad libitum*. Group I served as normal control, administered with distilled water; groups II, III, IV, and V were orally administered crude extract dissolved in distilled water at 5 mg/kg BW, 50 mg/kg BW, 300 mg/kg BW and 2000 mg/kg BW by intragastric route using a gavage method. The doses were selected according to OECD guideline No. 423 (OECD, 2001). Wellness parameters of animals were observed continuously during the first 30 min, 60 min, and periodically for the next twenty-four hours and then daily for fourteen days. All observations were systematically recorded.

The animals were fasted overnight prior to being sacrificed under anaesthesia using ketamine (150 mg/kg) and xylazine (15 mg/kg). Blood from each animal of all groups were collected via cardiac puncture using a 3 ml syringe with 26G ×$ \frac{1}{2} $ (0.45×13 mm) needle for biochemical analyses; alanine aminotransferase (ALT), aspartate aminotransferase (AST), alkaline phosphatase(ALP), blood urea nitrogen (BUN), uric acid (UA), creatinine (Cr) and total protein (TP). The remaining whole blood was collected in EDTA tubes for haematological parameters. The organs were examined for gross pathological changes and representative samples from the liver and kidney were collected in freshly prepared 10% buffered formalin for microscopic histopathology examination by cutting sections of 4–5µm thickness and stained with haematoxylin and eosin stain (Kadagi et al., 2014).

### Statistical analysis

Results were expressed as mean ± standard error of the mean (SEM). One-way analysis of variance (ANOVA) was used for data with normal distribution (body weight, ALP, ALT, AST, TP, Cr, urea, uric acid, HGB, RBC, MCH, RDW, WBC and platelets), while Kruskal-Wallis was used for data with non-normal distribution (HCT, MCHC and MCV). IBM SPSS software version 22 (Chicago, USA) was used to analyse all data. Statistical significant was considered at (*p* < 0.05).

## Results

**Figure 1:**
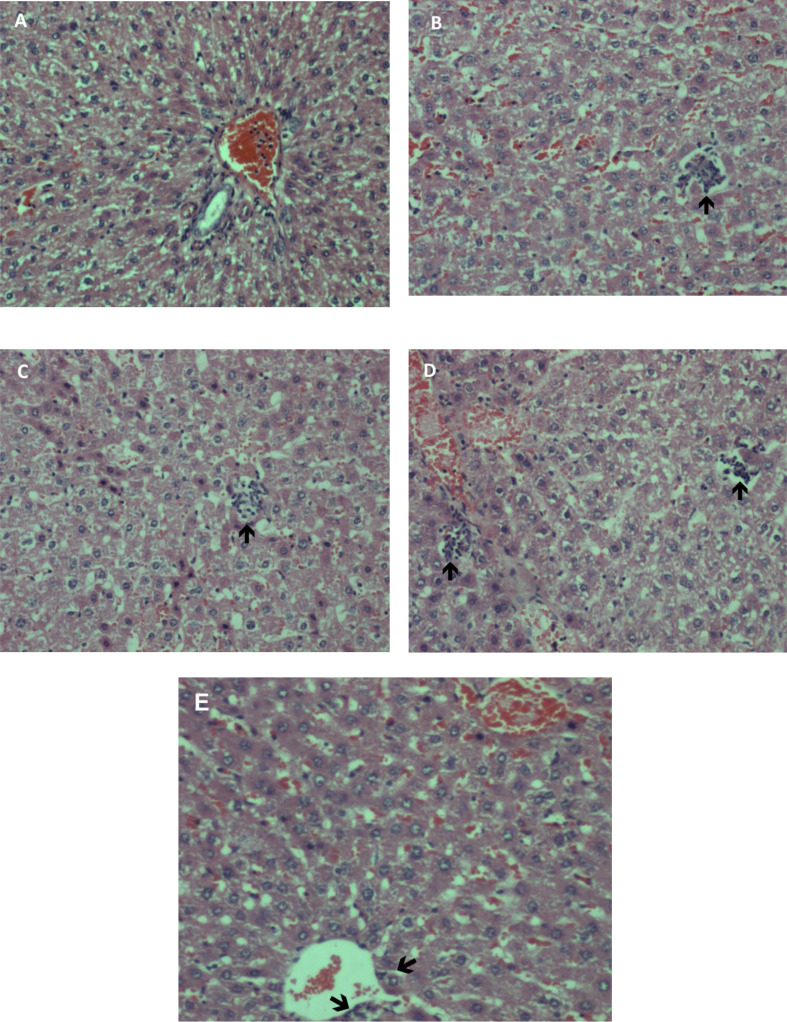
(A–E): Photomicrographs of liver sections in rats treated with methanolic extract of *A. africanus* (H&E stain, magnification ×100). (A): Control (dH_2_O). Liver, with occasional binucleation. No bile stasis or any steatosis seen. Central veins appeared normal and portal tracts were unremarkable. (B): 5 mg/kg BW, (C): 50 mg/kg BW and (D): 300 mg/kg BW. Liver showed mildly swollen hepatocytes, with occasional binucleation. The black arrows [→] indicates lobular hepatitis. Venules and portal tracts were unremarkable. (E): 2,000 mg/kgBW. Liver showed mildly swollen hepatocytes, occasional binucleation and normal central veins. Portal tracts were unremarkable.

**Figure 2:**
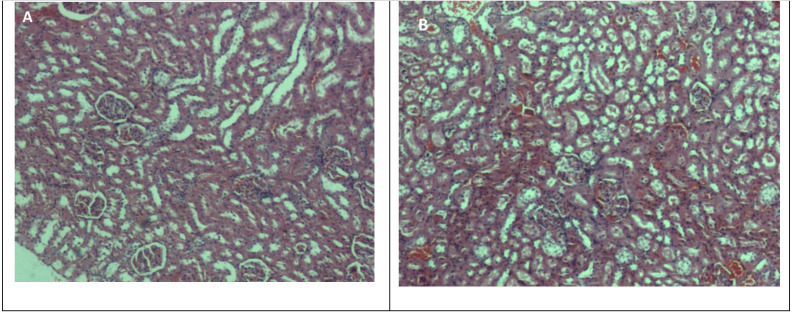
Photomicrographs of kidney sections in rats treated with methanolic extract of *A. africanus* (H&E stain, magnification ×40). (A) Control (dH_2_O) and (B) 2,000 mg/Kg BW. Both groups had kidneys exhibiting normal glomeruli, tubules and blood vessels.

The results obtained are summarised in Tables 1–4, which include the effects of methanolic extract on body weight, organ weight, serum biochemistry and haematology parameters. Figures 1 and 2 show photomicrographs of liver and kidney of treated and untreated rats with methanolic extract of *A. africanus*.

**Table 1:** Effect of MEAA on the body weight of treated and untreated rats.

**Treatment dose**	**Initial bodyweight (g)**	**Final body weight (g)**	**Mean body weight increased(g)**	**Weight gain(%)**
Control 5.0 ml Dil H_2_O	190.90 ± 5.75	239.65 ± 21.35	48.75	25.55
5.0 mg/kgBW MEAA	195.47 ± 9.84	228.59 ± 17.73	33.12	16.97
50 mg/kgBW MEAA	204.65 ± 2.97	241.50 ± 6.94	36.28	17.67
300 mg/kgBW MEAA	211.30 ± 2.97	246.75 ± 11.12	35.45	17.37
2,000 mg/kgBW MEAA	204.27 ± 5.19	243.18 ± 12.87	39.91	19.63

**Notes.**

Values are expressed as mean ± SEM, *n* = 3 for each group.

**Table 2:** Effect of (MEAA) on organs weight of treated rats.

**Treatment dose**	**Final body weight(g)**	**Liver weight(g)**	**Kidney weight(g)**
Control 5.0 ml Dil H_2_O	239.65 ± 21.35	9.64 ± 0.11	2.35 ± 0.24
5.0 mg/kgBW MEAA	228.59 ± 17.73	9.64 ± 0.22	1.99 ± 0.14
50 mg/kgBW MEAA	241.50 ± 6.94	10.95 ± 0.57	2.16 ± 0.03
300 mg/kgBW MEAA	246.75 ± 11.12	12.03 ± 1.51	2.47 ± 0.21
2,000 mg/kgBW MEAA	243.18 ± 12.87	11.92 ± 1.30	2.36 ± 0.12

**Notes.**

Values are expressed as mean ± SEM, *n* = 3 for each group.

**Table 3:** Effect of MEAA on biochemical indices of the treated and untreated rats.

**Treatment dose**	**ALT (U/L)**	**ALP (U/L)**	**AST (U/L)**	**Cr (µmol/L)**	**Urea (µmol/L)**	**Uric acid(µmol/L)**	**Total protein(gm/L)**
Control 5.0 ml DW	63.30 ± 0.33	158.00 ± 5.29	131.33 ± 13.90	22.66 ± 0.33	7.03 ± 0.20	115.66 ± 40.37	58.33 ± 3.38
5.0 mg/kgBW MEAA	72.50 ± 2.59	156.00 ± 6.92	212.00 ± 50.80	23.00 ± 0.57	7.05 ± 0.28	96.00 ± 31.75	56.33 ± 1.15
50 mg/kgBW MEAA	71.66 ± 6.83	209.66 ± 33.38	144.00 ± 24.24	22.33 ± 0.33	6.83 ± 0.38	62.00 ± 4.04	57.66 ± 0.88
300 mg/kgBW MEAA	69.33 ± 5.45	169.00 ± 12.22	131.00 ± 35.11	23.00 ± 0.57	6.53 ± 0.37	66.00 ± 17.69	59.33 ± 2.02
2,000 mg/kgBW MEAA	57.66 ± 5.54	172.33 ± 3.75	104.00 ± 16.44	22.33 ± 0.88	6.267 ± 0.26	53.33 ± 6.69	58.33 ± 1.20

**Notes.**

Values represent mean ± SEM, *n* = 3. Kruskal–Walis test was used to analyse the data and *P* < 0.05 was considered as statistically significant.

ALT Alanine aminotransaminase ALP Alkaline phosphatase AST Aspartate aminotransferase Cr Creatinine MEAA methanolic extract of *Asparagus africanus*

**Table 4:** Effect of MEAA on haematological parameters of the treated and untreated rats.

**Treatment dose**	**HGB (g/L)**	**HCT (L/L)**	**RBC (10**^**12**^**/L)**	**MCV (fl)**	**MCH (pg)**	**MCHC (g/L)**	**RDW (%)**	**WBC (10**^**9**^**/L)**	**Platelet (10**^**9**^**/L)**
Control 5.0 ml Dil H_2_O	137.00 ± 1.0	0.44 ± 0.01	6.93 ± 0.01	64.33 ± 1.15	19.8 ± 0.1	303.67 ± 0.57	12.33 ± 0.15	10.10 ± 0.1	756 ± 1.0
5.0 mg/kgBW MEAA	136.67 ± 1.15	0.44 ± 0.01	6.66 ± 0.09^a^	64.67 ± 0.58	19.87 ± 0.58	311.33 ± 1.15^*^	12.2 ± 0.17	11.13 ± 0.12^**^	564.67 ± 0.58
50 mg/kgBW MEAA	139.67 ± 4.73	0.44 ± 0.01	7.02 ± 0.32	62.0 ± 1.0	19.6 ± 0.2	318.33 ± 3.51^*^	11.73 ± 0.32^a^	12.00 ± 0.7^**^	977.67 ± 87.39^**^
300 mg/kgBW MEAA	143.33 ± 2.52^a^	0.44 ± 0.02	7.44 ± 0.04^a^	63.67 ± 1.53	19.33 ± 0.15	313.66 ± 1.53^*^	12.5 ± 0.2	11.23 ± 0.15^**^	1,014 ± 2.0^**^
2,000 mg/kgBW MEAA	132.0 ± 5.57^a^	0.42 ± 0.02	6.71 ± 0.53	61.0 ± 2.0	19.4 ± 0.43	312 ± 1.73^*^	11.6 ± 0.61^a^	8.33 ± 0.21^**^	817.67 ± 156.86

**Notes.**

Values are expressed as mean ± SEM, *n* = 3. One-way ANOVA was used to analyse the data and *P* < 0.05 was considered as statistically significant. Tukey HSD post-hoc test was further used to precisely determine the actual significance between groups.

RBC Red Blood Cell WBC White Blood Cell PCV Pack Cell Volume MCV Mean Corpuscular Volume MCH Mean Corpuscular Haemoglobin MCHC Mean Corpuscular Haemoglobin Concentration HCT Haematocrit RDW Red Cell Distribution Width PLT platelet Count MEAA methanolic extract of *Asparagus africanus* MAA methanolic extract of *A. africanus*

* *p* < 0.05 significantly different from control.

** *p* > 0.001 significantly different from control.

a *p* < 0.05 significantly different from each other in a column.

### Effect of MEAA on body weight gain and organs weight

The average weight gain of the treated groups was lower than that of the control group, with 5 mg/Kg BW being the lowest (Table 1). However, the difference in the percentage of weight increment in the control and the treated groups were statistically not significant. Table 2 shows no significant difference in the body and organs weight between control and treated groups

### Effect of MEAA on biochemical parameters

Methanolic extract treated groups showed no significant difference in all biochemical parameters such as ALT, ALP, AST, Cr, urea, uric and total protein (*p* > 0.05), between control and the treated groups (Table 3).

### Effect of MEAA on haematological parameters

Methanolic extract activity on MCHC showed a significant increase (*p* < 0.05) between control group and all treated groups. Furthermore, the platelet counts of 50 mg/kg BW and 300 mg/kg BW treated groups showed a significant increase (*p* < 0.001) when compared to the control group. In addition, RDW parameter demonstrated a significant increase in 50 mg/kg BW and 2,000 mg/kg BW treated groups (*p* < 0.05) (Table 4). HGB results for 300 mg/kg BW and 2,000 mg/kg BW groups were statistically increased significantly (*p* < 0.05). Likewise, for RBC results, 5 mg/kg BW group were also statistically significant between groups (*p* < 0.05). Meanwhile, HCT, MCV and MCH did not show any statistical significance (*p* > 0.05) amongst the various groups.

### Histopathology

Liver sections of rats treated with methanolic extract of *A. Africanus* at 5 mg/Kg BW, 50 mg/Kg BW, 300 mg/Kg BW and 2,000 mg/Kg BW showed congestion. Mildly swollen hepatocytes with occasional binucleation and focal lobular hepatitis were also observed. No bile stasis or steatosis were seen, venules and portal tracts were unremarkable (Figs. 1B–1E). For kidney sections, both control and treated groups even with the highest dose at 2,000 mg/Kg BW have normal glomeruli, tubules, interstitium and blood vessels (Figs. 2A–2B).

## Discussion

In this study, the laboratory processed dried form of *A. africanus* was extracted using methanol, with the percentage yield of 9.6% of the initial weight. This resulting yield of extract was better than that previously reported by Moshi et al. (2003) where it was observed that the dry product of extraction yielded only 1–2% of initial material. Acute toxicity study is invariably the first step at initial stage in almost all screening investigation of herbal medicine of unknown potential. Most acute toxicity data are then used to predict safe dose limit in clinical application because some herbs exert harmful effects even at very low doses to sensitive tissues and organs in the body. Hence, multiple dose studies are considered as one of the most valuable tools used in evaluating the biosafety profile of medicinal plants (Abiodun, Oyinlade & Oluwatobi, 2015; Woode & Abotsi, 2011).

In this study, root extract of *A. africanus* was administered at doses of 5 mg/kg BW, 50 mg/kg BW, 300 mg/kg BW and 2,000 mg/kg BW according to OECD guideline 423 (OECD, 2001). Findings showed that there were no abnormal changes in the general appearance and no alteration in the behaviour of animals in all treated groups. In addition, zero mortality rate was also recorded even in rats treated with the higher dose of *A. africanus* (2,000 mg/kg BW). The mean percentage weight gain of rats in untreated control group was 25.55%, which is higher than the reported weight gain in treated groups, this could explain the traditional uses of this plant as anti-diabetic and anti-obesity agent. This result is in line with reports of Okolie (2014), in which *A. africanus* among many plants have been screened for the anti-diabetic property and has shown to be effective in blood sugar reduction to significant level (Okolie, 2014). In our study, the effect of methanolic extracts on biochemical indices revealed a non-significant increase in ALP and decrease in uric acid. Alkaline phosphatase (ALP) is an enzyme that mainly released into blood circulation by the liver and bones and higher concentrations may be as a result of the mild liver damage caused by *A. africanus*. Histopathology findings of the liver confirmed this result and showed only congestion with mildly swollen hepatocytes and occasional binucleation. On the other hand, uric acid is a product of the metabolic breakdown of purine nucleotides and an increase in the excretion of uric acid from the blood into the urine can lead to lower blood concentrations of uric acid. Nonetheless, further investigation is needed to study the exact mechanism on how root extract of *A. africanus* can lead to hypouricemia. Other serum biochemical parameters such as ALT, AST, Cr, Urea and total protein were not affected.

Conversely in the present study, a significant increase was observed in some of haematology parameters, such as MCHC (*p* < 0.05), WBC and platelets (*p* < 0.001) when compared between treated groups and control. The increment in WBC and platelets in animals treated with *A. africanus* extract may suggest that the plant administration could induce inflammatory reaction such as inflammatory bowel disease, however further studies are needed to confirm this adverse effect. Inflammatory conditions such as inflammatory bowel disease has been reported to cause increment in platelets (Kuter, 2019), which may be due to the effect of plant extract. This result is in contrary to the data obtained from oral administered of *Abrus precatorius* of aqueous leaves extract to rats for 21 days, which showed a significant decrease (*p* < 0.05) in the levels of red blood cells, haemoglobin, packed cell volume and platelets in groups treated with 200 and 400 mg/kg BW of the extract (Wakawa, Adejo & Fasihuddin, 2015). However, literature have shown that the administration of medicinal plants can alter the normal ranges of haematological parameters (Attal et al., 2010).

Meanwhile, HGB, RBC and RDW parameters showed significant differences amongst the treated groups, whereas HCT, MCV and MCH were not statistically significant (*p* > 0.05) as compared to control group. The interpretation would be that methanolic root extract of *A. africanus* affected haematology parameters as opposed to the effects of other popular herbs such as *Azadirachta indica* (Ashafa, Orekoya & Yakubu, 2012). Histopathology examination was conducted according to Theise (2007). Liver and kidney appeared normal and showed no markedly difference between control and treated rats. However, the livers of rats treated with methanolic extract showed congestion with mildly swollen hepatocytes, occasional binucleation and focal lobular hepatitis, but no bile stasis or steatosis. *A. africanus* methanolic extract did not cause any death or abnormal behaviour effects in the treated rats.

The presence of different phytochemicals in medicinal plants can be linked to several biological activities and alterations in the biochemical parameters and histology patterns of the organs, especially on the liver and kidney. Phytochemical studies revealed that *A. africanus* contained several organically active metabolites such as polyphenols, saponins, phenolic glycosides, phytosterols and terpenes were identified in roots part of the plant of *A. africanus* (Abera, Ashebir & Basha, 2019). These findings are in line with our phytochemical analysis of methanolic extract of *A. africanus* which revealed the presence of steroidal saponins such as sarsasapogenin and stigmasterol (El-Ishaq, Alshawsh & Chik, 2019a). This saponins is most implicated bioactive phytochemical for its oestrogenic properties and anti-implantation activities, which could be also alter some of the haematological parameters. The presence of saponins and other non-steroidal compounds in *A. africanus* might have different pharmacological and physiological effects on animal tissues or might act synergistically to produce different biological and toxicological effect (Kebede et al., 2016). Furthermore, Okello, David & Oloro (2019) have revealed the presence of anti-fertility bioactive compounds such as saponins, alkaloids and phenolic in *A. africanus* root extract. In addition, the same study concluded that *A. africanus* was non-toxic up to the maximum dose of 12.8 g and the anti-fertility activity of the aqueous crude extract was found to be dose dependent.

The effective doses of *A. africanus* have been reported in the literature and varies from one study to another depends on the experimental model. As example, the antifertility of aqueous root extract of *A. africanus* in Albino rats has been identified at 300 mg/kg BW (Geremew, Yalemtsehay & Eyasu, 2006), while the anti-conception dose in Wister rats has been reported at 300 mg/kg BW (Okello, David & Oloro, 2019). The analgesic and anti-inflammatory effective dose of *A. africanus* in Swiss albino rat has been confirmed at doses less than 1,000 mg/kg BW (Kim et al., 2009). Another study has suggested the anti-inflammatory dose of *A. africanus* in Swiss albino rat is 1,264 mg/kg BW (Hassan et al., 2012). All these pharmacological effective doses are still less than the maximum safe dose (2,000 mg/kg). Since *A. africanus* is commonly used in traditional medicine and can be investigated further as a potential source of useful bioactive compounds for several reproductive uses in human, therefore our study can be used as basis for the safety profile of this plant. The present study has some limitations such as the test was only carried out using crude extract and investigated in one animal species. The isolation of the active components in the extract will be the basis for future studies on the effect of this extract on uterine tissue and other organs.

## Conclusion

The results from this study showed that *A. africanus* methanolic extract caused no mortality, no behavioural abnormality and no significant biochemical alteration in rats, although methanolic extract modulated some of the haematology parameters. Therefore, it could be deduced that extract will probably be safe up to 2,000 mg/kg BW.The obtained result could be used to justify the safety of traditional application of the plant for treatment of various ailments, however further studies are needed to confirm these pharmacological activities and to investigate the underlying mechanism of actions of the isolated bioactive constituents of this plant.

##  Supplemental Information

10.7717/peerj.9138/supp-1 Supplemental Information 1 Methanolic extract raw data

10.7717/peerj.9138/supp-2 Supplemental Information 2 SPSS output for methanolic extra raw data

## Additional Information and Declarations

### Data Availability

The following information was supplied regarding data availability: Raw data is available as Supplemental Files.
